# Structural insights into the nirmatrelvir-resistant SARS-CoV-2 M^pro^ L50F/E166A/L167F triple mutant-inhibitor-complex reveal strategies for next generation coronaviral inhibitor design

**DOI:** 10.1039/d5md00356c

**Published:** 2025-08-15

**Authors:** Conrad Fischer, Jimmy Lu, Marco J. van Belkum, Sydney Demmon, Pu Chen, Chaoxiang Wang, Tayla J. Van Oers, Tess Lamer, M. Joanne Lemieux, John C. Vederas

**Affiliations:** a Department of Chemistry, University of Alberta Edmonton AB T6G 2G2 Canada john.vederas@ualberta.ca; b Department of Biochemistry, Li Ka Shing Institute of Virology, University of Alberta Edmonton T6G 2E1 AB Canada

## Abstract

Drug-resistance is an eminent threat in antiviral therapy, and is currently a concern in nirmatrelvir-based therapy of SARS-CoV-2. Nirmatrelvir (antiviral component in Paxlovid) binds covalently to the active site cysteine of the main protease of SARS-CoV-2 (M^pro^), thereby blocking enzyme activity and halting viral replication. *In vitro* passage experiments mimicking a multi-dosage nirmatrelvir treatment regime, identified M^pro^ variants with mutations in the active site and near the C-terminal dimerization interface with variable levels of nirmatrelvir resistance. One such variant harbors a triple mutation in M^pro^, L50F/E166A/L167F, that displays decreased potency for nirmatrelvir (IC_50_ ∼ 850–1600 nM) and ibuzatrelvir while viral replication remained similar to that of the wildtype (WT) virus. We here confirm a previously developed short peptide aldehyde bisulfite compound 4 as potent inhibitor for SARS-CoV-2 M^pro^ L50F/E166A/L167F and related variants. A co-crystal structure reveals tight inhibitor binding that is stabilized by a network of hydrogen bonds formed by the mutated residues A166 and F167. This study provides the groundwork for optimized M^pro^ inhibitors against potential emerging variants of SARS-CoV-2, as well as strategies for broad-spectrum inhibitor design against variants of M^pro^.

## Abstract 2

4 was confirmed as low nanomolar binder of several coronavirus main proteases, including a nirmatrelvir-resistant variant of concern (L50F/E166A/L167F). A co-crystal structure informs about future design strategies for pan-coronaviral inhibitors.

## Graphical abstract

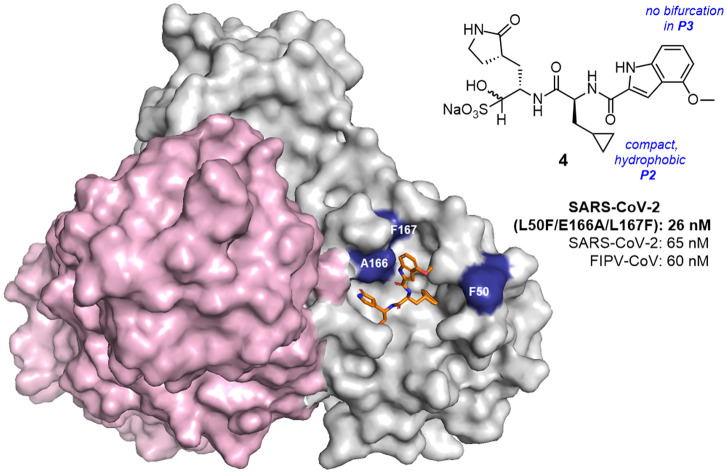

## Introduction

Nirmatrelvir is a selective protease inhibitor in Pfizer's anti-COVID drug Paxlovid, the first line of treatment for COVID-19 infections with potential to progress to a severe state^1^ as well as long-term COVID patients.^2^ It binds covalently in a reversible fashion to the active site cysteine of the main protease (M^pro^) of SARS-CoV-2, a chymotrypsin-like protease, and inhibits viral polyprotein processing that ultimately halts viral replication. Nirmatrelvir pressure has been shown to inflict proximal and active-site mutations in M^pro^ of SARS-CoV-2 which reduces drug potency and contributes to Paxlovid resistance.^3–5^ Similar resistance to first-line antiviral drugs has been observed against influenza^6^ and hepatitis C^7^ further highlighting this alarming health threat and the need for immediate action to identify alternative treatment regimes. One particularly concerning M^pro^ mutation that emerged from passage experiments of the wildtype (WT) SARS-CoV-2 virus in the presence of nirmatrelvir, is the M^pro^ triple mutant L50F/E166A/L167F.^5^ It possesses two mutations near the S1 sub-pocket of the active site (E166A/L167F) that increase its hydrophobicity and reduce its thermostability, as well as a distal mutation (L50F) near the dimerization interface. This triple mutant displays about 70-times higher IC_50_ values with nirmatrelvir, while maintaining WT-like fitness in cellular and *in vivo* animal models.^3–5^ The distal mutation may compensate for reduced substrate binding affinity in the active site caused by the double mutation (E166A/L167F) thereby restoring viral viability.^8^ Although rare, omicron SARS-CoV-2 lineages with these three mutations have been identified from patient isolates and have been circulating since December 2021, further supporting the *in vitro* data.^9^ An inhibitor-bound M^pro^ L50F/E166A/L167F crystal structure has not yet been reported, likely due to the reduced binding affinity of nirmatrelvir to this mutant enzyme, and therefore structural evident to support this hypothesis is currently lacking. Here, we assessed the IC_50_ values of a panel of previously reported peptide-based SARS-CoV-2 M^pro^ inhibitors with the mutant enzyme M^pro^ L50F/E166A/L167F and identified a lead inhibitor with an approximately 30-fold improvement in inhibition compared to nirmatrelvir. For this we synthesized and used an optimized, longer FRET substrate to more reliably determine kinetic parameters, enabling us to identify compound 4 as a low nanomolar M^pro^ L50F/E166A/L167F-binder. Crystallization of M^pro^ L50F/E166A/L167F and 4 led to the formation of single crystals at 1.9 Å that highlight inhibitor-induced stabilization of conformation flexibility of the active site as consequence of altered surface charge.

## Results & discussion

### Modified FRET peptide provides a more robust measurement of WT and mutant SARS-CoV-2 M^pro^ catalytic efficiency

**Scheme 1:**
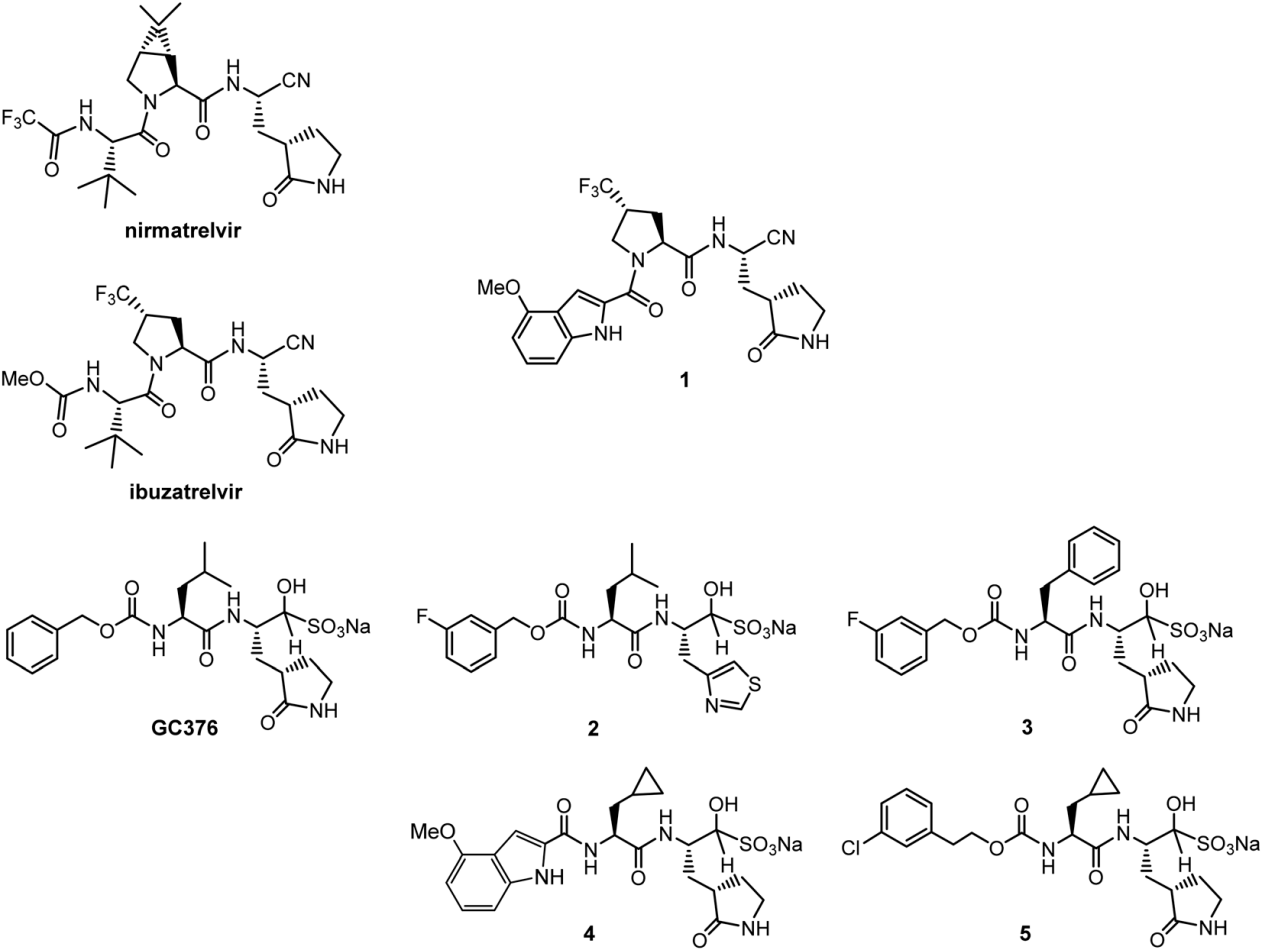
Compounds studied in this paper (1–5) were derived from literature-developed anti-coronaviral drugs nirmatrelvir, ibuzatrelvir, and GC376. The synthesis of compounds 2,^16^4,^17^ and 5,^17^ is described in the literature, those of compounds 1 and 3 can be found in the SI.

The L50F/E166A/L167F triple mutation in SARS-CoV-2 M^pro^ is the first reported main protease mutation that confers resistance to nirmatrelvir, the active agent in Paxlovid™, when selecting for drug resistant mutations *in vitro*. To evaluate the potency of known SARS-CoV-2 M^pro^ inhibitors for the M^pro^ triple mutant in comparison to the WT protease, we first determined IC_50_ values of nirmatrelvir, the improved second-generation candidate ibuzatrelvir, and GC376, a known anti-coronaviral peptide aldehyde derivative (Scheme 1). The M^pro^ triple mutant L50F/E166A/L167F was cloned and expressed using a SUMO-fusion tag that, upon cleavage, releases the native enzyme in a traceless fashion. Cleavage of the SUMO-fusion tag allows the mutant enzyme to dimerize and become fully active, in a similar fashion to the WT M^pro^ as described previously (Fig. S1).^10^ Forster-resonance energy transfer (FRET) assays with designed FRET peptide substrates are one of the most common ways to assess M^pro^ activity and success of inhibitors. However, recent reports indicate relatively slow processing of commonly used FRET substrates by certain M^pro^ mutants, complicating the determination of precise IC_50_ values.^5,11^ Thus, we first focused on the identification of a more physiological FRET substrate for use in future inhibition assays with the triple mutant enzyme. We initially determined steady state kinetics for the triple mutant using our in-house FRET substrate 2-Abz-SVTLQ↓SGY^(NO_2_)^R-OH, which is a hybrid substrate modeled after the nsp4–nsp5 and nsp5–nsp6 cleavage sites.^12^ Compared to the WT M^pro^, this substrate's processing by the triple mutant is noticeably slower, as reflected by a *K*_M_ = 320 μM and *k*_cat_ = 0.26 s^−1^ (Table 1), likely due to poorer fit into the substrate pocket. This results in a ∼35-times lower catalytic efficiency of the M^pro^ triple mutant compared to the WT M^pro^ (∼800 M^−1^ × s^−1^*vs.* ∼28 000 M^−1^ × s^−1^, Table 1, Fig. S2). This is in contrast with single and double mutants of the SARS-CoV-2 M^pro^ which display only a modest reduction of enzymatic activity with classic FRET substrates, usually no more than 1-fold.^8,13,14^ A recent kinetic study of the 11 M^pro^ cleavage sites suggests the N-terminal nsp4–nsp5 cleavage sequence as the primary site of M^pro^ catalysis, as it displayed the highest catalytic efficiency relative to the other 10 cleavage sites.^15^ Therefore, we synthesized a longer substrate, as commonly used in the literature with main protease FRET assays^12,14^ to test if it would be better recognized by the triple mutant enzyme, leading to lower *K*_M_ and higher *k*_cat_ values. Building off the known Dabcyl-KTSAVLQ↓SGFRKME(Edans)-NH_2_ substrate, solely containing the nsp4–nsp5 cleavage sequence, we synthesized a 15-mer FRET substrate incorporating an N-terminal 2-Abz group and 3-nitrotyrosine as quencher (substituting for Phe, Fig. S3) and compared its catalytic performance with the M^pro^ triple mutant and WT M^pro^. While the overall catalytic efficiency of this longer substrate does not significantly change, it has a lower *K*_M_ with the M^pro^ triple mutant (*K*_M_ = 250 μM, Table 1, Fig. S2), compared to the shorter substrate, suggesting of a higher affinity. Consequently, we chose this substrate as the FRET reporter for our herein reported IC_50_ assays.

**Table 1:** Kinetic parameters of investigated FRET substrates *vs.* M^pro^ WT and M^pro^ L50F/E166A/L167F

Substrate	WT	WT	L50F/E166A/L167F	L50F/E166A/L167F
	Dabcyl-KTSAVLQ↓S GFRKME(Edans)-NH_2_	2-Abz-SVTLQ↓SGY^(NO_2_)^R-OH	2-Abz-SVTLQ↓SGY^(NO_2_)^R-OH	2-Abz-KTSAVLQ↓SGY^(NO_2_)^RKME-OH
*K*_M_ (M)	4.6 × 10^−6^ (lit. 23)	80 × 10^−6^ (lit. 10)	3.2 × 10^−4^	2.5 × 10^−4^
*k*_cat_ (s^−1^)	0.29 ± 0.03 (lit. 23)	2.25 ± 0.1 (lit. 10)	0.26 ± 0.05	0.19 ± 0.04
*k*_cat_/*K*_M_ (M^−1^ × s^−1^)	(0.26 ± 0.05) × 10^6^ (lit. 23)	(0.28 ± 0.05) × 10^5^ (lit. 10)	(0.82 ± 0.06) × 10^3^	(0.77 ± 0.05) × 10^3^

### Modified GC376 analog displays sub-nanomolar IC_50_ inhibition towards the SARS-CoV-2 wild-type, as well as L50F/E166A/L167F triple mutant M^pro^

While IC_50_ values of nirmatrelvir, ibuzatrelvir, and GC376 with the triple mutant M^pro^ are generally higher compared to the WT M^pro^, GC376 showed moderate inhibition (IC_50_ = 200 nM), which is 4- to 5-times lower than the IC_50_ values of nirmatrelvir (IC_50_ = 850 nM) and ibuzatrelvir (IC_50_ = 1080 nM), respectively (Table 2, Fig. S4). It is worth noting, the IC_50_ value for nirmatrelvir is slightly lower than the reported value by Westberg and co-workers,^11^ likely due to our use of a longer FRET substrate for measuring kinetic parameters. Exchange of the aliphatic P3 portion in ibuzatrelvir for a 4-methoxy indole moiety, which previously demonstrated retained or improved potency of respective WT M^pro^ inhibitors, renders compound 1 inactive for L50F/E166A/L167F. As GC376 retained moderate inhibition, we then explored how exchange of the P1, P2, and P3 group would affect binding potency for the triple mutant. Substitution of the P1 2-oxopyrrolidine ring for an aromatic thiazol-4-yl group (compound 2), which has recently been disclosed as an alternative SARS-CoV-2 M^pro^ inhibitor,^16^ does not significantly alter binding potency for L50F/E166A/L167F. On the other hand, exchange of the P2 leucine residue for a larger phenylalanine residue (compound 3), renders the inhibitor inactive. However, installation of a less bulky cyclopropyl group preserves inhibition potency as observed with compounds 4–5. Surprisingly, an aromatic 4-methoxy indole moiety is accepted in P3 position without compromising activity of compound 4, which has, of all tested inhibitors, the lowest IC_50_ value (IC_50_ = 26 nM), with *K*_i_ data (Fig. S7) supporting this finding. In previous studies we demonstrated both 4 and 5 as nanomolar M^pro^ inhibitors and antivirals against SARS-CoV-2 and feline infectious peritonitis virus (FIPV), responsible for the viral replication of a fatal viral infection affecting cats.^17,18^ Comparing the structure–activity relationship of the tested inhibitors it becomes clear that L50F/E166A/L167F is more selective for an inhibitor conformation that avoids bifurcation of the inhibitor molecule towards both S3 and S4, a prominent feature for both nirmatrelvir^19^ and ibuzatrelvir^20^ bound to the active site cavity of WT M^pro^. Furthermore, a prospective lead inhibitor should possess additional donor/acceptor groups to maximize binding interaction with the native and mutated protease. These two features seem best represented in compound 4.

**Table 2:** IC_50_ values of studied inhibitors with SARS-CoV-2 M^pro^ WT and SARS-CoV-2 M^pro^ L50F/E166A/L167F in comparison with available literature IC_50_ data of these inhibitors with other coronaviral M^pro^ targets

IC_50_ values			
	SARS-CoV-2 M^pro^ L50F/E166A/L167F IC_50_ (nM)	SARS-CoV-2 M^pro^ IC_50_ (nM)	FIPV M^pro^ IC_50_ (nM)
Nirmatrelvir	850 ± 40	25 ± 5	n.a.
Ibuzatrelvir	1080 ± 50	30 ± 10	n.a.
GC376	200 ± 15	115 ± 15	130 ± 20 (lit. 18)
1	>2 × 10^5^	90 ± 15	n.a.
2	480 ± 25	160 ± 20	n.a.
3	>2 × 10^5^	440 ± 30	n.a.
4	26 ± 5	65 ± 10	60 ± 20 (lit. 18)
5	130 ± 25	80 ± 20	50 ± 20 (lit. 18)

### Crystal structure of compound 4 in complex with SARS-CoV-2 M^pro^ L50F/E166A/L167F reveal differences in hydrogen bond interactions that lead to altered activity

**Fig. 1:**
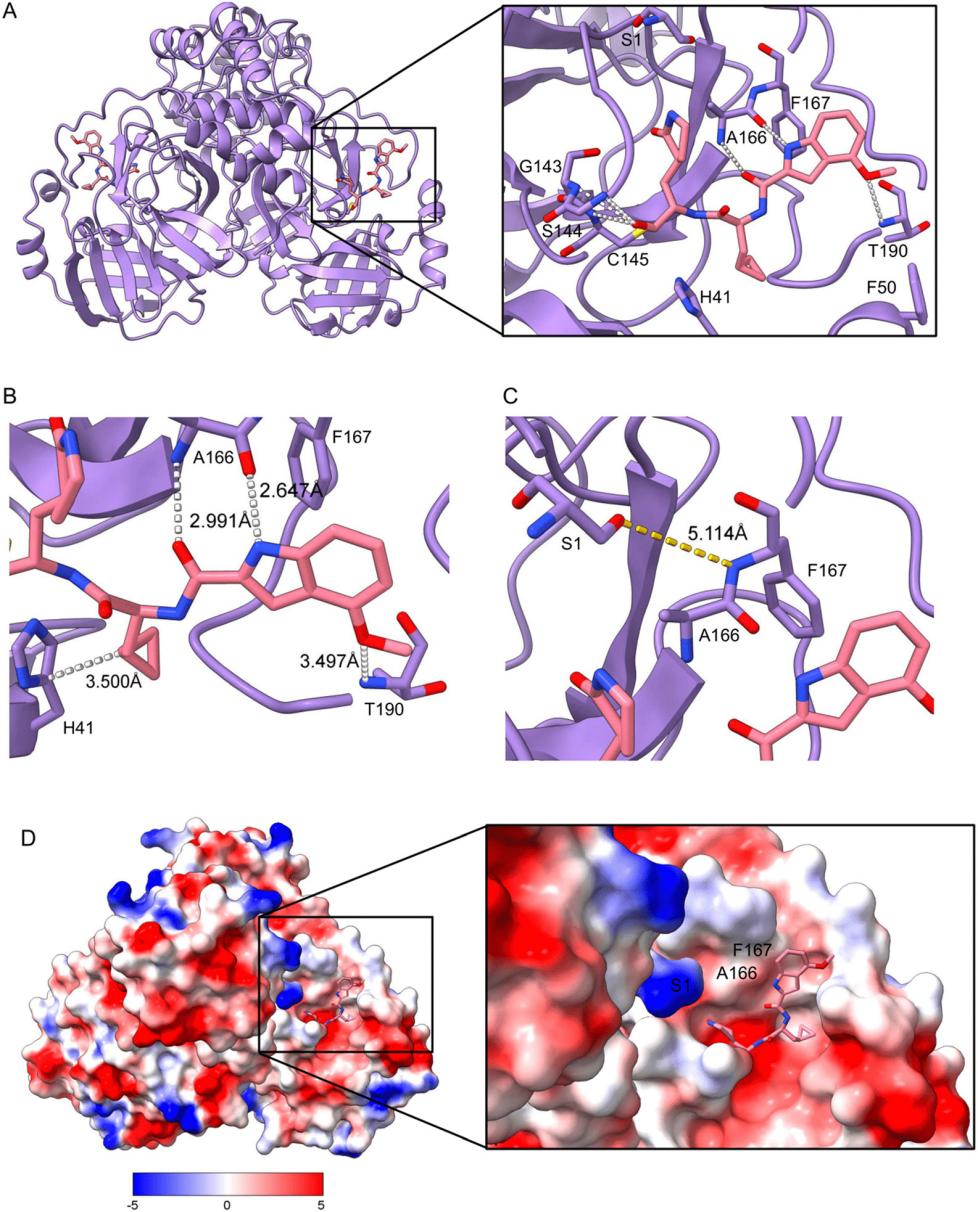
Crystal structure of SARS-CoV-2 L50F/E166/L167F in complex with compound 4, (PDB code 9N3M). A) Overall domain organization of M^pro^ L50F/E166/L167F-inhibitor complex reveals a homo-dimer organization. B) The P3 position of compound 4 forms a hydrogen bond network (white dashed lines) with residues within the active site. C) A166 is unable to form a hydrogen bond with Ser1 of the other protomer (highlighted by a yellow dashed-line indicating an O–H(N) distance of 5.114 A unsuitable for a valid hydrogen bond). D) Surface charge within the active site shows a neutral charge at the S3/4 position.

To confirm our hypothesis, we performed co-crystallization of the triple mutant with compound 4. The resulting structure complex was solved to 1.9 Å in the *P*2_1_2_1_2 space group (Fig. 1A, Table S1). Here, we see the P3 4-methoxy indole fits in the S3/S4 position of the M^pro^ active site where it is stabilized by hydrogen bond coordination with the backbone amide of A166 (Fig. 1B). The P1 2-oxopyrrolidine also forms weak hydrogen bonds with residues G143 and S144, adjacent to the active site cysteine. Upon further observation, the E166A mutation alters the packing of the M^pro^ dimer. In WT SARS-CoV-2 M^pro^, the carboxylate group of E166 from protomer A forms a hydrogen bond with the NH group of Ser1, referred to as the “N-finger”, from protomer B.^21^ This interaction plays a role in the formation of the S1 pocket for both protomers in each dimer and facilitates the binding of substrates and inhibitors at the S1 site. When the E166A mutation is present, the N-finger is unable to form a hydrogen bond with A166 resulting in a dimer that is less stable relative to its wild-type (Fig. 1C), further supported by reduced stability when measured using differential scanning fluorometry (*T*_m_ 49.5 °C *vs.* 59.7 °C, Fig. S5). The dynamic nature of the triple mutant M^pro^ is further highlighted when comparing the inhibitor complex with the recently disclosed apo-form of the triple mutant M^pro^ (PDB: 8U25)^22^ where we see binding of compound 4 causes movement of the loop containing the E166A and L167F mutations that might rationalize the significant decrease in thermostability compared to WT M^pro^ (Fig. S6). Additionally, with a small, non-polar methyl group in place of a polar carboxylate group, surface charge of the S1 pocket is lost and becomes more hydrophobic (Fig. 1D). The L50F mutation is also located adjacent to the active site, forming the entrance of the substrate binding groove. The phenylalanine causes widening of the S2 pocket, as previously observed in the single mutation,^24^ causing a shift towards a more open conformation. The L50F mutation has also recently been reported to facilitate interactions that form a “dimer of dimers”, where the authors suggest the F50 of one dimer compensates for reduced enzymatic activity caused by the E166A/L167F mutation through protein–protein interactions with the opposing dimer's active site.^22^ While their hypothesis is intriguing, it cannot be inferred from crystal packing how this protease will behave in a physiological setting, as no evidence of a dimer of dimers has been reported in nature.

**Fig. 2:**
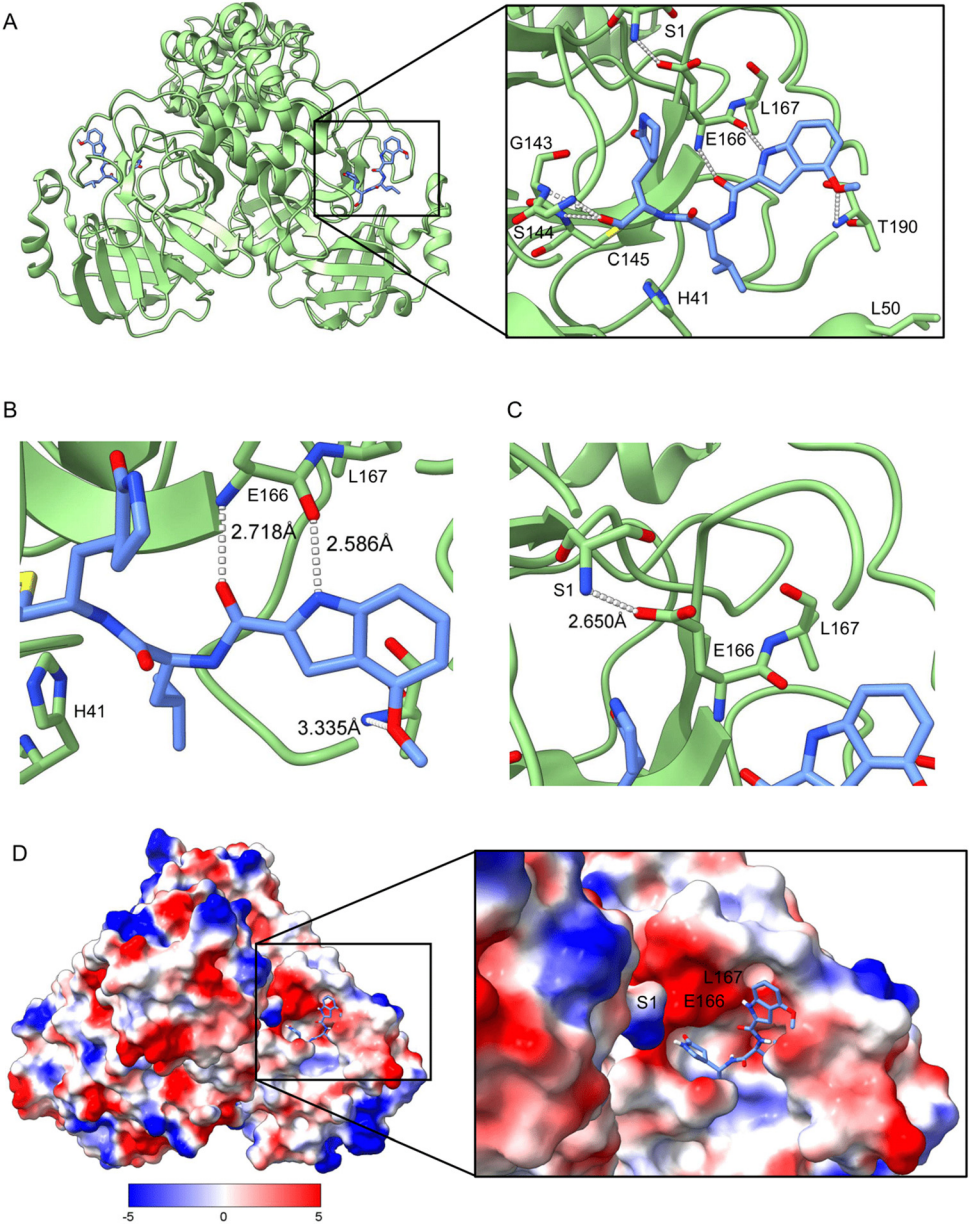
Crystal structure of WT SARS-CoV-2 M^pro^ in complex with an inhibitor that closely resembles compound 4 (PDB code 7LDL). A) Overall domain organization of WT M^pro^-inhibitor complex reveals a homo-dimer organization. B) The P3 position of compound 4 forms a hydrogen bond network with residues within the active site. C) E166 forms a hydrogen bond with Ser1 of the other protomer, referred to as the N-finger. D) Surface charge within the active site shows a positive charge at the S3/4 position.

To further evaluate how compound 4 fits in the active site of the triple mutant protease, we compared the structure with a co-crystal structure of the WT M^pro^ in complex with a related inhibitor (Fig. 2A). This inhibitor only differs by a leucine instead of a cyclopropyl alanine residue in P2, thereby lacking the hydrophobic packing with H41 observed with compound 4 (Fig. 1B and 2B). As expected, the Ser1 from one monomer forms a hydrogen bond with residue E166 from the other, stabilizing the dimer (Fig. 2C). A lack of hydrogen bonding from the N-finger and altered surface charge in the active site compromises the fit of natural substrates and substrate mimetics, such as FRET substrates, leading to the observed slower catalytic processing of FRET substrates in the triple mutant compared to the WT enzyme.

## Conclusions

The development of broad spectrum antivirals against emerging SARS-CoV-2 and related coronaviral variants is an urgent objective for pandemic preparedness. Herein we demonstrate that a peptide aldehyde bisulfite 4, previously made by our group, is a potent inhibitor of the triple mutant L50F/E166A/L167F SARS-CoV-2 main protease, a variant of concern that is resistant to nirmatrelvir and ibuzatrelvir. Unlike nirmatrelvir, compound 4 binds tightly to the mutant, WT, and closely related coronavirus M^pro^ with low nanomolar IC_50_. A co-crystal structure highlights a flat, extended aromatic P3 group, the lack of bifurcation towards S3, and a small cyclopropyl alanine P2 group as beneficial structural features to ensure tight and universal binding. The L50F/E166A/L167F triple mutation confers structural features that destabilize M^pro^ dimerization and alter its ability to bind current inhibitors used to treat SARS-CoV-2 that include nirmatrelvir and ibuzatrelvir. Our results suggest that inhibitors engineered in P2 and P3 to induce fit in the active site mutated M^pro^ variants constitute a viable strategy for the design of broad-spectrum next generation inhibitors for coronaviral diseases including SARS-CoV-2.

## Materials and methods

### Synthesis and purification of FRET substrates

Amino acid sequences of employed FRET substrates are disclosed in Fig. S2 and were synthesized by solid phase peptide synthesis (SPPS). The synthesis of the 10-mer FRET has been described earlier.^10^ All commercially available reagents and protected amino acids were purchased and used without further purification unless otherwise noted. All the solvents used for reactions were used without further purification unless otherwise noted. Dry solvents refer to solvents freshly distilled over appropriate drying reagents prior to use. For the synthesis of the 15-mer FRET the first amino acid (R) was loaded as follows: 2-chlorotrityl chloride resin was transferred to a SPPS vessel and washed with dry CH_2_Cl_2_ (2 × 10 mL) and then dry DMF (2 × 10 mL) for one min each, and then bubbled under Ar in dry DMF (10 mL) for 10 min. Fmoc-Arg(Pbf)-OH (1.0 equiv., based on desired resin loading) and DIPEA (5.0 equiv.) were suspended in 10 mL of a 50/50 mixture of dry CH_2_Cl_2_/DMF. This solution was bubbled under Ar for 2.5 h to load the desired amino acid onto the solid support, continually topping up the CH_2_Cl_2_ to maintain an approximately 10 mL volume. To end cap any remaining trityl groups, dry MeOH was added to the vessel (0.8 mL per gram of resin) and bubbled under Ar for 15 minutes. After draining, the resin was washed with dry DMF (3 × 10 mL), dry CH_2_Cl_2_ (3 × 10 mL), and again DMF (3 × 10 mL). The resin was elongated by coupling 3 equiv. of Fmoc-protected amino acid, 3 equiv. of PyBOP (benzotriazol-1-yloxytripyrrolidinophosphonium hexafluorophosphate), 3 equiv. of HOBt (hydroxybenzotriazole) and 9 equiv. of DIPEA (*N*,*N*-diisopropyl ethylamine) in DMF for 1 hour. Fmoc residues were deprotected using a 20% solution of piperidine in DMF (3 × 7 min). The N-terminal 2-Abz building block was attached as Boc-protected amino acid using the same conditions as for Fmoc-protected amino acid couplings. To cleave the mature peptide, resin-bound analogue was suspended in 95/2.5/2.5 TFA/TIPS/H_2_O with shaking for 4 h. The resin was removed *via* filtration through glass wool, rinsed with TFA, and the solution concentrated *in vacuo*. Cold diethyl ether (2 × 5 mL) was added to triturate the crude residue. The diethyl ether was decanted and briefly centrifuged for 3 minutes at 13 000 rpm to pellet any residual peptide. The ether was removed, and the peptide pellet was then dried thoroughly by centrifugation in a vacuum centrifuge for 5 minutes. The pellet and triturated crude residue were pooled together and dissolved in 0.1% aqueous TFA. FRET peptide was purified using a Vydac Si C18 RP-HPLC semi-preparative column (300 Å, 5 μM, 10 × 250 mm) with aqueous 0.1% TFA (solvent A) and 0.1% TFA in acetonitrile (solvent B) as eluents. The analytical purification method used was: 0–3 min 10% B, 3–4.5 min 10–25% B, 4.5–14.5 min 25–40% B, 14.5–17 min 40–90% B, 17–19.5 min 95% B, 19.5–20.5 min 95–10% B, 20.5–30 min 10% B. The HPLC fractions were pooled and lyophilized to produce the peptides as a light-yellow powder. Purity of the 15-mer was confirmed with HPLC and HRMS (SI, Fig. S3) using an Agilent Technologies 6130 LCMS with a core–shell C8-column (1.7 μm, 100 Å, Phenomenex Kintex). A solvent gradient (A: H_2_O with 0.1% TFA, B: ACN with 0.1% TFA) was employed as follows: 0–5 min, 2–100% B; 5–8 min, 100% B.

### Cloning, expression, and purification of M^pro^ proteins

The cloning of the M^pro^ genes of SARS-CoV-2 and FIPV, and the expression and isolation of these M^pro^ enzymes have been described before.^10,17,18^ For the SARS-CoV-2 M^pro^ triple mutant L50F/E166A/L167F gene, DNA was obtained from Genscript, and the sequence (Genbank: MN908947.3) was adjusted to incorporate the three L50F/E166A/L167F mutations and codon optimized for expression in *Escherichia coli*. The gene was cloned into the pET SUMO vector (Invitrogen) in such a way that the M^pro^ protein is in frame with the His-tagged SUMO protein. The resulting gene was expressed in *E. coli* BL21(DE3) at 32 °C for 5 h. The M^pro^ triple mutant was isolated and purified as described before.^25^ Briefly; cells were harvested, suspended in lysis buffer (20 mM Tris-HCl pH 7.8, 150 mM NaCl) and lysed by sonication. Cell debris was spun down by centrifugation (27 000*g* for 20 min at 4 °C) and after addition of 5 mM imidazole, the supernatants were loaded onto a Ni-NTA resin column (Qiagen). The resin columns were washed with 10 column volumes of lysis buffer containing 20 mM imidazole and the fusion protein was eluted with lysis buffer containing 300 mM imidazole and 1 mM dithiothreitol (DTT). After the protein sample was dialyzed against lysis buffer containing 1 mM DTT, 1 mM EDTA, and 0.002% Tween-20, the fusion protein was digested with His-tagged SUMO protease (McLab) for 2 h at 4 °C to remove the SUMO tag. The protein mixture was subsequently loaded onto a Ni-NTA resin column and the M^pro^ L50F/E166A/L167F was obtained in the flow-through. The M^pro^ L50F/E166A/L167F was further purified using size exclusion chromatography (Sephadex G-15, GE Healthcare), with buffer containing 20 mM Tris-HCl pH 7.8, 150 mM NaCl, 1 mM DTT, 1 mM EDTA, and 0.002% Tween-20 and concentrated using an Amicon Ultra-15 filter with a MWCO of 10 kDa.

### General procedure for enzymatic assays

All assays were analysed with a Spectramax i3x microplate reader controlled by Softmax Pro software (version 6.5.1, Molecular Devices). Readings were taken in black 96-well flat bottom polypropylene microplates (Corning) under specific time regimes (see below) at 37 °C in assay buffer (20 mM bis-Tris, pH 7.6, 1 mM DTT, 0.02% Tween-20). Excitation and emission wavelength specific for 2-Abz were set to 320 nm (bandwidth 9 nm) and 420 nm (bandwidth 15 nm), respectively. Initial rates were fit to the linear portion of the reaction progress curve, accounting for less than 10% substrate hydrolysis.

### Steady state enzyme kinetics

Michaelis–Menten kinetics were measured in 20 mM bis-Tris buffer (pH 7.6), containing 1 mM DTT and 0.02% Tween-20 in a total volume of 160 μL per well. 200 nM enzyme was used in the assay with FRET substrate concentrations ranging from 20 to 2500 μM. Reactions were initiated by addition of enzyme and fluorescence read every minute for 60 minutes using above settings. After correcting values for photo-bleaching and inner filter effect progress curves in RFU s^−1^ were converted into μM s^−1^ with the help of a calibration curve constructed with the fluorescent N-terminal cleavage fragment 2-Abz-SVTLQ (WT) or 2-Abs-KTSAVLQ (L50F/E166A/L167F), respectively. Initial velocities were calculated from the linear curve part (first 20 minutes) and plotted against FRET concentrations to obtain values of *K*_M_ and *v*_max_ using the non-linear, least squares regression analysis in Graphpad Prism 9 software. To calculate *k*_cat_, *v*_max_ was divided by the molar concentration of enzyme used in each assay (as specified above). With these values of *k*_cat_ and *K*_M_, the value of *k*_cat_/*K*_M_ was subsequently calculated assuming a fixed amount of active enzyme used in the experiment.

### IC_50_ determination

Various concentrations of each inhibitor (final concentrations 2 × 10^5^–0.3 nM) in assay buffer (20 mM bisTRIS, pH 7.8, 1 mM DTT, 0.5% DMSO) were pre-incubated with SARS-CoV-2 M^pro^ or 3 M protease (this was done with a final concentration of 80 nM 200 nM in assay with similar values obtained) for 30 min at 37 °C. After addition of the FRET substrate (final concentration 300 μM), fluorescence response was read every 30 s for 10 min (WT-M^pro^) and 60 min (L50F/E166A/L167F), respectively. Difference of fluorescence (ΔRFU s^−1^) of initial linear curve points (first 90 s) were used for IC_50_ calculations. Data were fitted and plotted using Prism (sigmoidal 4PL, version 9, PrismGraphPad, USA). All measurements were done in triplicate and values are presented as mean ± standard error.

### Differential scanning fluorometry

6 μM of triple mutant M^pro^ with a final concentration of 5× for SyproOrange dye (Thermo Fisher Scientific, USA) in 50 mM Tris-HCl, pH 8.0, 150 mM NaCl. All samples were run in duplicate. The thermal scan was conducted from 25 to 95 °C, at 0.5 °C min^−1^ (ViiA 7 Realtime PCR System, ThermoFisher). Compound 4 was added to the triple mutant M^pro^ in a molar ratio of 1 : 1 and incubated for 30 minutes prior to reading. The melting point (*T*_m_) was calculated by fitting the raw fluorescence data over the temperature using the Boltzmann equation in GraphPad Prism program (Prism 9, GraphPad Software, USA).

### Protein crystallography and data refinement

Purified SARS-CoV-2 M^pro^ triple mutant (L50F/E166A/L167F) was dialyzed against 20 mM Tris-HCl pH 7.8, 150 mM NaCl, 1 mM DTT overnight at 4 °C. The protein was concentrated using an Amicon Ultra-15 filter with a MWCO of 10 kDa to 8 mg mL^−1^ and incubated in 4 molar excess of compound 4 for 2 hours at 4 °C. The protein-inhibitor complex was subjected to the JCSG plus and PACT crystallization screen (Molecular Dimensions) with the best crystals optimized in 0.22 M potassium thiocyanate and 20% w/v PEG3500 in a 1 : 1 ratio using hanging drop vapor diffusion at room temperature. Crystals were incubated with 20% glycerol as a cryoprotectant, prior to freezing. Data collection took place at Stanford Synchrotron Radiation Lightsource (SSRL) (Menlo Park, CA) beamline 12-2 with Blu-Ice using the Web-Ice interface and at Canadian Light Source, Inc.

Diffraction data sets were collected at 100 K in a cold nitrogen stream using SSRL beamline 12-2 at a wavelength of 0.979460, equipped with an Eiger 16 M Pixel Array detector. X-ray detector Software (XDS) and was used for processing of the data sets. The structure was determined by molecular replacement, with the crystal structure of the free enzyme of the SARS-CoV-2 M^pro^ (PDB entry 6WTM) as a search model, using the Phaser program from Phenix, version v1.19.2–4158. Refinement was performed with phenix.refine in Phenix software. Statistics of diffraction, data processing and model refinement are given in Table S1. The model was inspected with Ramachandran plots and final models displayed using UCSF ChimeraX molecular graphics software version 1.9.

## Author contributions

CF, JCV, and MJvB conceptualization; CF, MJvB and JCV methodology; CF, CW, TJVO, and TL and synthesis of FRET substrates; MJvB and MJL synthesis of M^pro^ enzymes; CF and TJVO investigation, analysis, and validation; JL, SD, and PC crystallization and structure solution; CF writing – original draft; CF, MJvB, TJVO, TL, JCV and MJL writing – review & editing.

## Conflicts of interest

There are no conflicts to declare.

## Supplementary Material

MD-OLF-D5MD00356C-s001

## Data Availability

Data for this article, including kinetic studies, IC_50_ data, and refinement statistics are available in SI. See DOI: https://doi.org/10.1039/D5MD00356C. Crystal structure of SARS-CoV-2 L50F/E166/L167F in complex with compound 4 is deposited in Protein Data Bank (PDB code 9N3M).^26^
