# Supplementary material for: Structural insights into the nirmatrelvir-resistant SARS-CoV-2 Mpro L50F/E166A/L167F triple mutant-inhibitor-complex reveal strategies for next generation coronaviral inhibitor design
Source: RSC Med Chem. 2025 Aug 15;16(10):5032–40. doi: 10.1039/d5md00356c (PMC12356143; doi:10.1039/d5md00356c)
Supplement: MD-016-D5MD00356C-s001 [file MD-016-D5MD00356C-s001.pdf]

Supplementary Information (SI) for RSC Medicinal Chemistry.
This journal is © The Royal Society of Chemistry 2025

Supplemental Information

Structural insights into the nirmatrelvir-resistant SARS-CoV-2 M^{pro} L50F/E166A/L167F triple mutant-inhibitor-complex reveal strategies for next generation coronaviral inhibitor design

Conrad Fischer[a], Jimmy Lu[b], Marco J. van Belkum[a], Sydney Demmon[b], Pu Chen[b], Chaoxiang Wang[a], Tayla J. Van Oers[a], Tess Lamer[a], Joanne Lemieux[b], John C. Vederas[a,*]

[a] Department of Chemistry, University of Alberta, Edmonton AB, T6G 2G2, Canada
[b] Department of Biochemistry, Membrane Protein Disease Research Group, University of Alberta, Edmonton AB, T6G 2R3, Canada

Table of Contents

Purification data of triple mutant SARS-CoV-2 M^{pro} 2
Determination of Steady State kinetics for 3M with different FRET substrates 3
Analytical data for 15-mer FRET substrate 3
IC_{50} data for various inhibitors with SARS-CoV-2 M^{pro} (WT) and SARS-CoV-2 M^{pro} 3M (L50F/E166A/L167F) 4
Data collection and refinement statistics 5
Differential scanning fluorometry 6
Structural comparison of apo-M^{pro} L50F/E166A/A167F vs inhibitor 4 bound complex 7
K_i data for various inhibitors with SARS-CoV-2 M^{pro} 3M (L50F/E166A/L167F) 8

Purification data of triple mutant SARS-CoV-2 M^{pro}

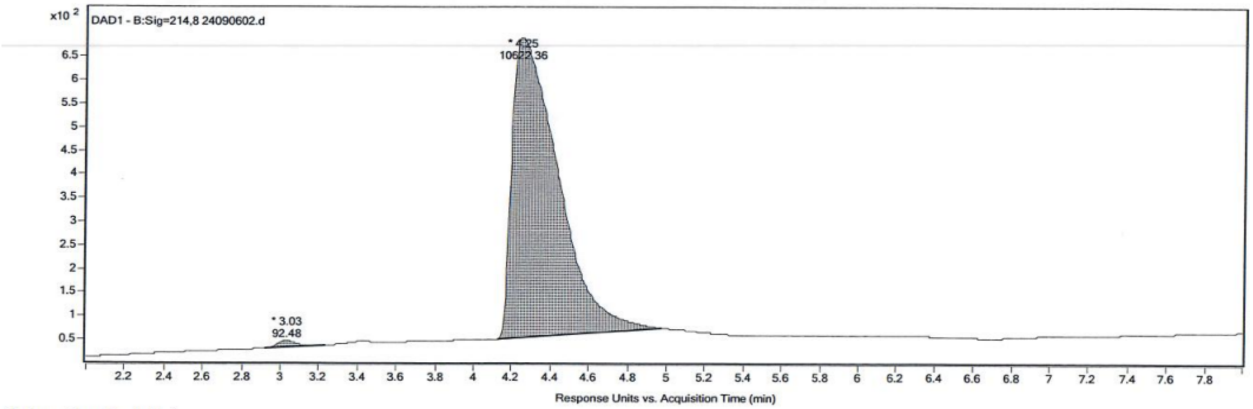

Integration Peak List

Peak	Start	RT	End	Height	Area	Area %
1	0.3	0.41	0.56	26.88	166.39	1.57
2	2.92	3.03	3.23	14.17	92.48	0.87
3	4.13	4.25	4.97	638.03	10622.36	100

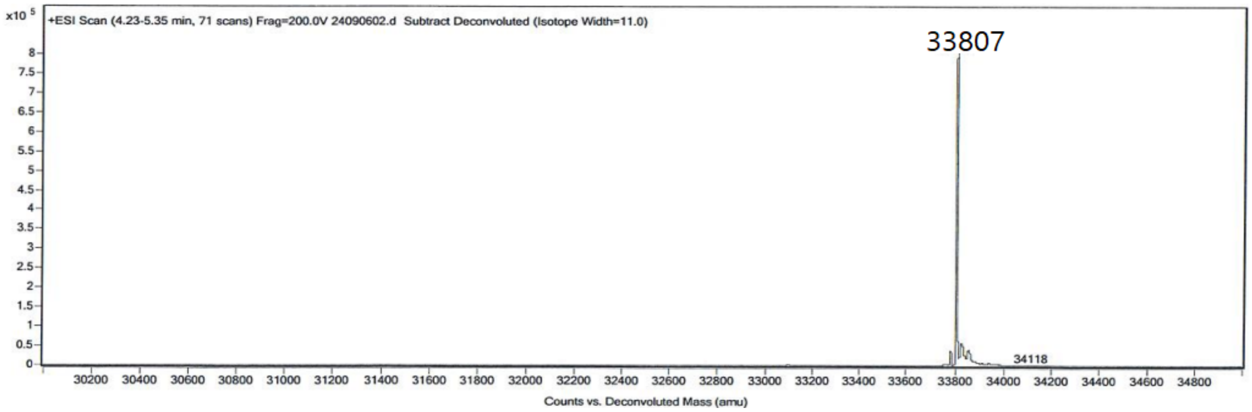

Figure S1. Chromatogram and de-convoluted mass-spectrum of SARS-CoV-2 3M confirming the mass of the SUMO-tag-free full protein (Mw = 33807 g/mol).

Determination of Steady State kinetics for 3M with different FRET substrates

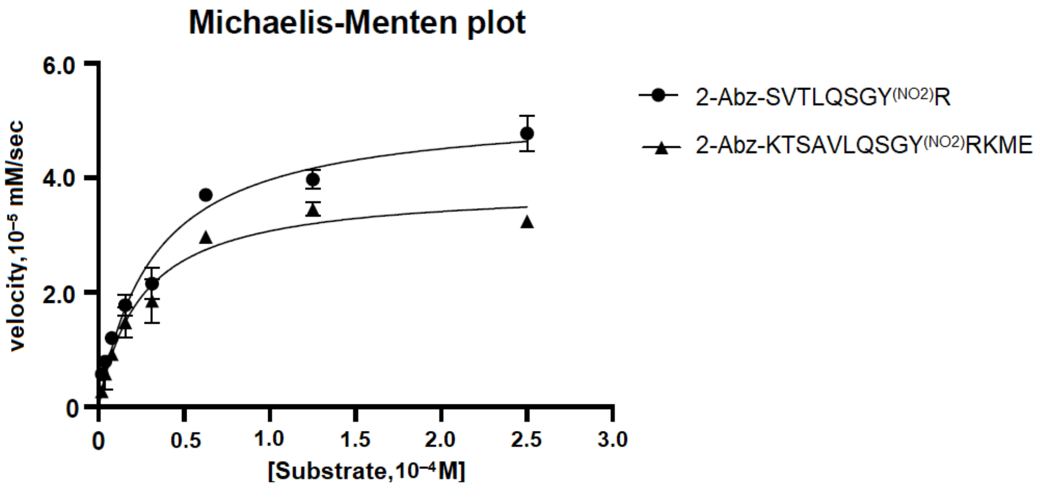

Figure S2. Michaelis-Menten plot for traditional shorter FRET (•) and newly developed 15-mer FRET substrate (▲). Data points are from triplicate measurements. Error bars are SEM.

Analytical data for 15-mer FRET substrate

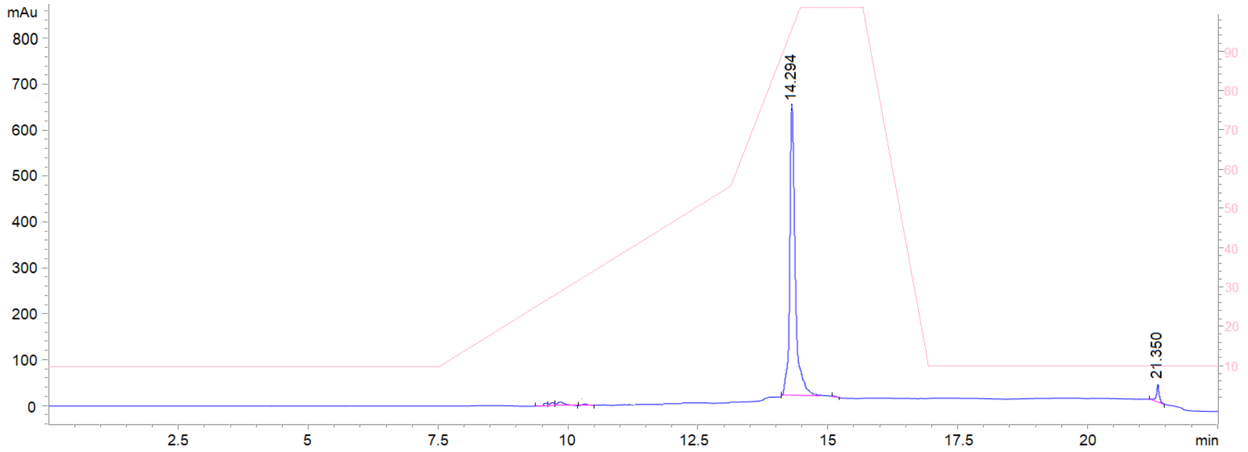

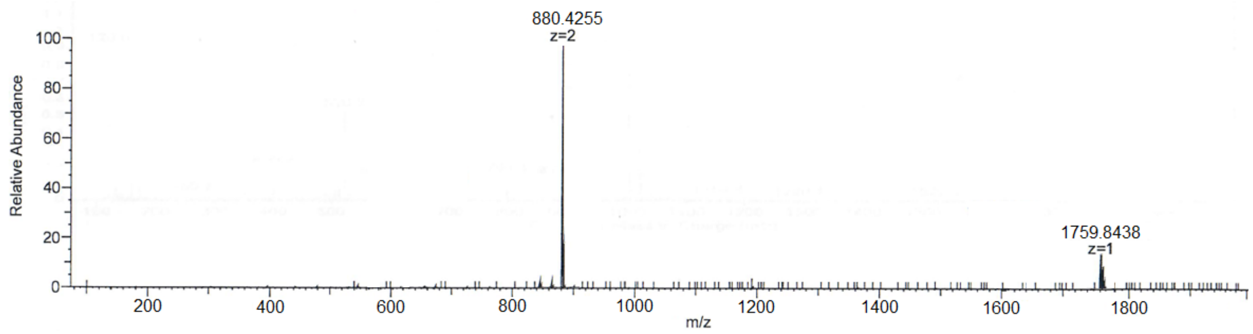

Figure S3. HPLC run and HRMS data of purified 15-mer FRET.

IC$_{50}$ data for various inhibitors with SARS-CoV-2 M^{pro} (WT) and SARS-CoV-2 M^{pro} 3M (L50F/E166A/L167F)

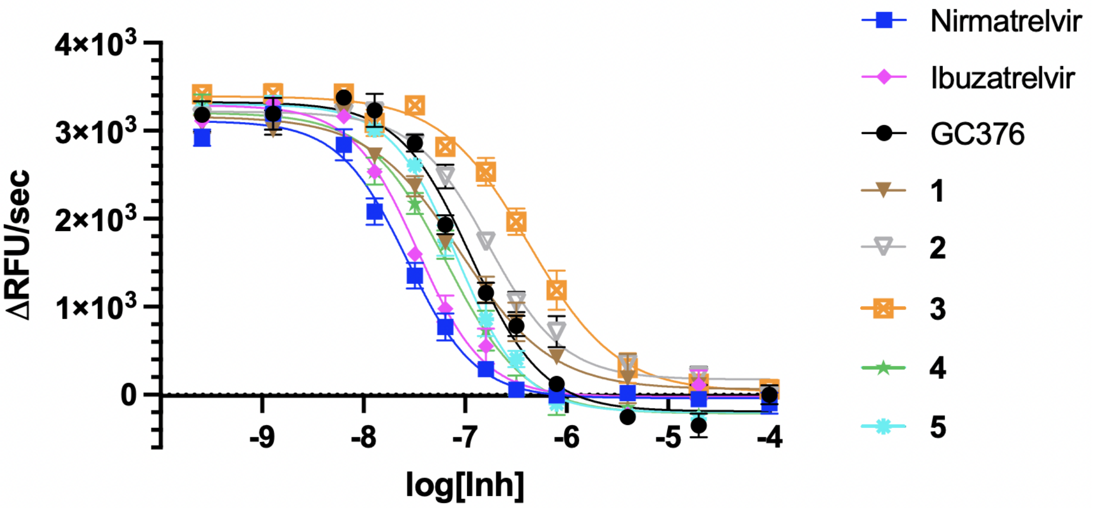

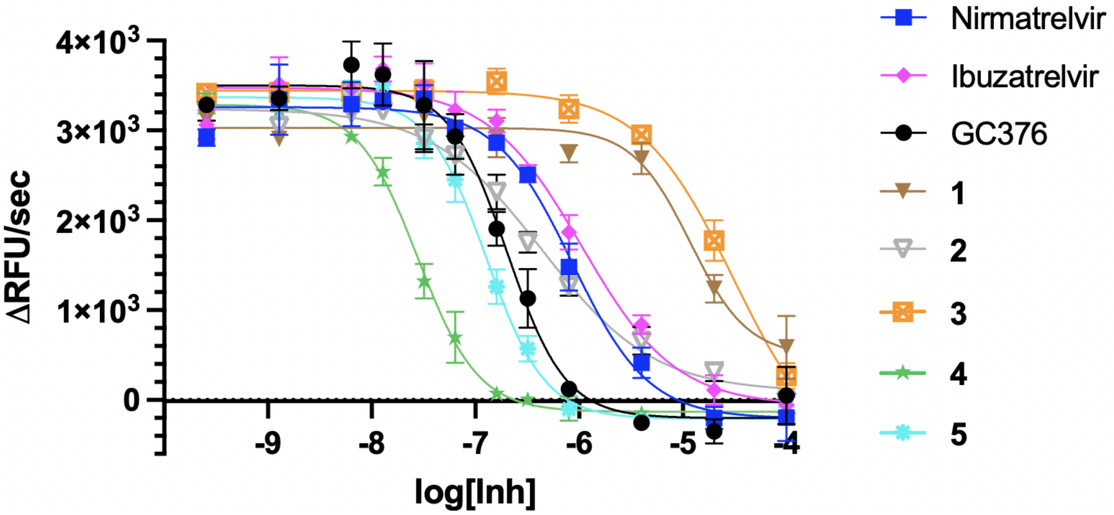

Figure S4. IC$_{50}$ graphs for tested inhibitors with wild-type SARS-CoV-2 M^{pro} (top) and triple mutant SARS-CoV-2 M^{pro} (L50F/E166A/L167F, bottom). Data points are from triplicate measurements. Error bars are SEM.

Data collection and refinement statistics

Supplementary Table 1: Data collection and refinement statistics (molecular replacement)

	L50F/E166A/L167F + **4**
PDB entry	9N3M
**Data collection**	
Space group	$P2_12_12$
Cell dimensions	
a, *b*, *c* (Å)	45.286, 64.467, 105.929
α, β, γ (°)	90, 90, 90
Resolution (Å)	41.64 - 1.9 (1.968 - 1.9)
Observations	233958 (23505)
R_{merge}	0.1259 (1.691)
$I / \sigma I$	9.75 (1.20)
Completeness (%)	99.66 (99.48)
Redundancy	9.3 (9.5)
CC1/2	99.60 (47.90)
**Refinement**	
Resolution (Å)	37.06 - 1.90
No. reflections	25106
R_{work} / R_{free}	20.73/24.17
No. atoms	2510
Protein	2371
Ligand/ion	32
Water	107
B-factors	52.51
Protein	52.55
Ligand/ion	49.16
Water	52.51
R.m.s. deviations	
Bond lengths (Å)	0.009
Bond angles (°)	1.11

*Values in parentheses are for highest-resolution shell. Each data set was collected from a single crystal.

Differential scanning fluorometry

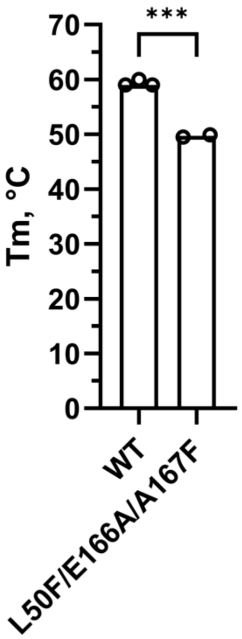

Figure S5. Supplemental Figure X. Differential scanning fluorometry analysis of the triple mutant M^{pro}, compared to the WT reveal differences in protein stability. ***: $p < 0.001$ between wild type and the mutant. T_m of WT M^{pro} was determined from Chen et al. 2023 using the same protocol.

Structural comparison of apo-M^{pro} L50F/E166A/A167F vs inhibitor 4 bound complex

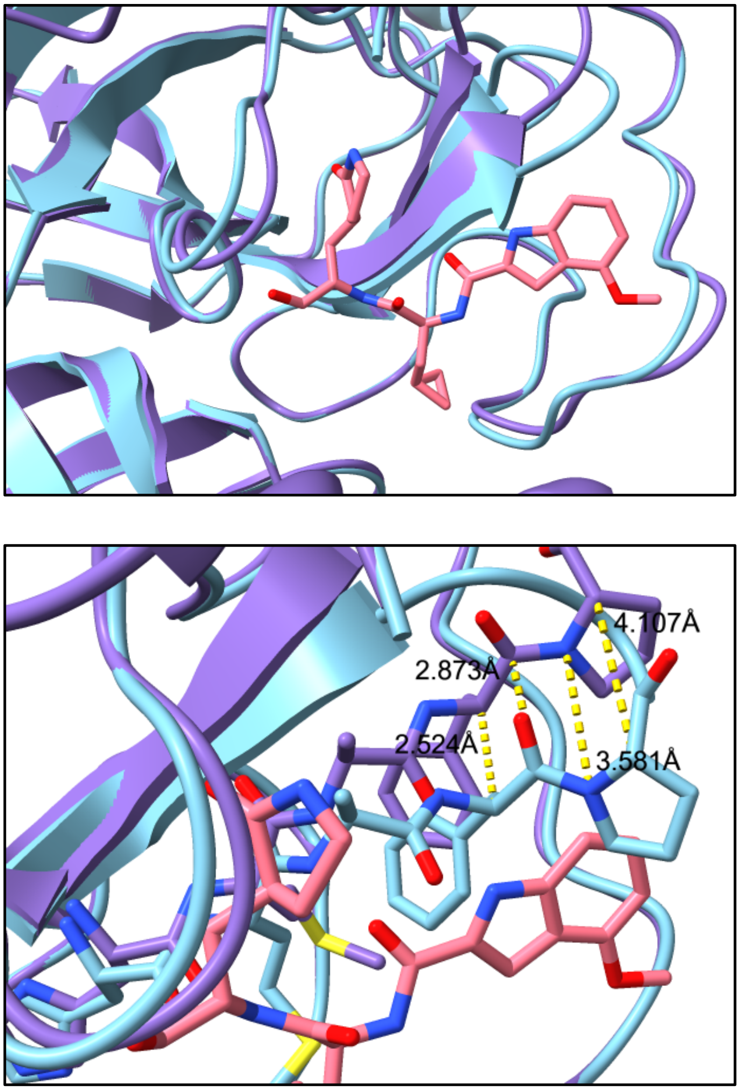

Figure S6. Structural comparison of apo-M^{pro} L50F/E166A/A167F (purple; PDB code 9N3M) to M^{pro} L50F/E166A/A167F bound to compound **4** (light blue; PDB code 8U25) reveals the active site is flexible and dynamic in order to accommodate the binding of **4.** The top panel is a cartoon representation while the bottom panel displays the stick residues to measure the distance moved by residues A166, F167 and P168.

K_i data for various inhibitors with SARS-CoV-2 M^{pro} 3M (L50F/E166A/L167F)

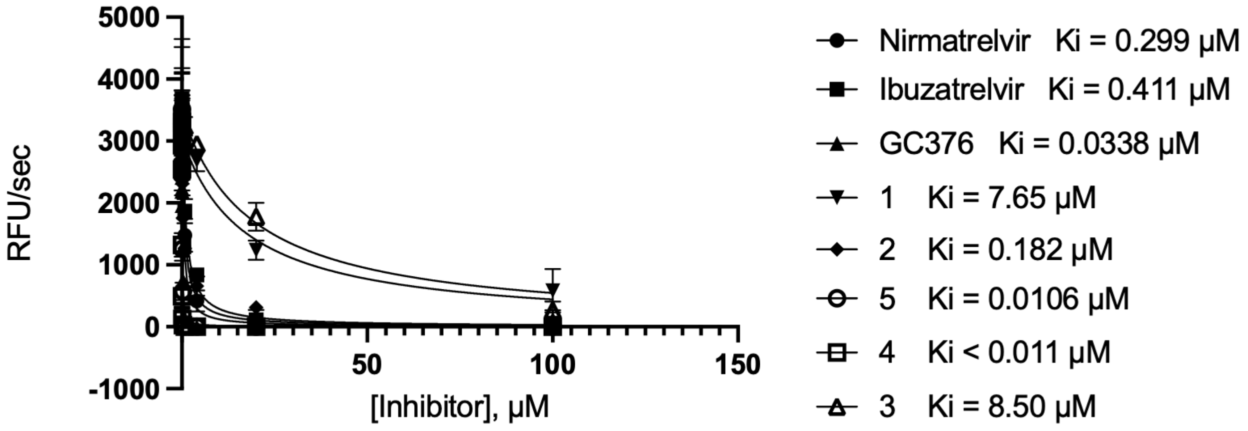

Figure S7. K_i graphs for tested inhibitors triple mutant SARS-CoV-2 M^{pro} (L50F/E166A/L167F). Data points are from triplicate measurements. Error bars are SEM. The K_i value for compound **4** was too low to be accurately determined in this assay.
